# *KRAS* status analysis and anti-EGFR therapies: is comprehensiveness a biologist's fancy or a clinical necessity?

**DOI:** 10.1038/sj.bjc.6605582

**Published:** 2010-02-16

**Authors:** E Lopez-Crapez, L Mineur, H Emptas, P-J Lamy

**Affiliations:** 1 Department of Clinical Biology, CRLC Val d'Aurelle-Paul Lamarque Cancer Institute, 208 rue des Apothicaires, Montpellier 34298, France; 2 Department of Medical Oncology, Sainte-Catherine Institute, Avignon, France; 3 Pathology Laboratory, Histosud, Le Pontet, France

**Sir,**

Both single-arm studies (Di Fiore *et al*, 2007; Lièvre *et al*, 2008) and randomised clinical trials (Amado *et al*, 2008; Karapetis *et al*, 2008) for the treatment of metastatic colorectal cancer (mCRC) with anti-EGFR monoclonal antibodies (mAbs) have unambiguously demonstrated that the occurrence of somatic *KRAS* mutations is a highly predictive marker of resistance to such therapies. Based on these data, analysis of *KRAS* mutation status in mCRC patients is now routinely carried out to select candidates for anti-EGFR treatment. However, it is still a matter of debate which *KRAS* codons should be investigated and which genotyping methods should be used.

To date, all of the reported studies focused on the analysis of missense *KRAS* mutations in codon 12 and 13. Indeed, substitutions at these codons (i) represent up to 90% of *KRAS* alterations found in colorectal cancer (Forbes *et al*, 2009), (ii) result in the accumulation of the protein in the active GTP-bound conformation (Malumbres and Barbacid, 2003) and (iii) impair the therapeutic effect of anti-EGFR mAb. Nevertheless, the recent data reported by Loupakis *et al* (2009) showed that rare mutations occurring at codon 61 or 146 are clinically relevant and predict resistance to cetuximab and irinotecan treatment. Furthermore, according to our personal experience, in a series of 845 surgical samples from mCRC patients, who were referred to our institution for diagnosis of *KRAS* mutations, the analysis by high-resolution melting (HRM) followed by bi-directional direct sequencing of exon 2 identified 307 (36.33%) mutated samples, among which 12 showed rare variants. Six tumours carried rare codon 13 substitutions (five c.37G>T, p.G13C and one c.37C>G, p.G13R). Four tumours presented double-point mutations (two patients had the c.38_39GC>AA, p.G13E modification, one patient the c.34_35GG>CT, p.G12A modification and one patient the c.34_35GG>TT, p.G12F modification) and two patients showed two uncommon *KRAS* mutations. The first one was an in-frame c.30_31 insGGA insertion resulting in a glycine insertion (p.G10_A11insG) in a rectal lesion located 7 cm from the anal verge of a 74-year-old man. Histological examination classified this tumour as well differentiated with a T3NxM1 stage. This uncommon mutation has only been previously reported in one CRC (Simi *et al*, 2008) patient and in a case of childhood myeloid leukaemia (Bollag *et al*, 1996). This duplication has been shown to induce cellular transformation *in vitro* and activation of the RAS–MAPK signalling pathway linked to impaired intrinsic GTP hydrolysis and resistance to GAPs (Bollag *et al*, 1996). Based on these data, the presence of this *KRAS* variant could be supposed to predict resistance to anti-EGFR therapies. The second uncommon *KRAS* mutation was a heterozygous 6-bp in-frame c.36_37insGCTGGT insertion that was responsible for the insertion of both one extra alanine and one extra glycine (p.G12_G13insAG). This mutation was found in a hepatic metastasis lesion from a 65-year-old man with a sigmoid colon cancer. To the best of our knowledge, this tandem duplication of both codon 12 and 13 has not been described before in any cancer type. Although the functional characterisation of this insertional variant is not available, previous *in vitro* data (Klockow *et al*, 2000) have reported that synthetic mutants showing insertions of extra amino acids in the *KRAS* codon 10–17 region (phosphate-binding loop) can promote cellular outgrowth and stimulate the MAP-kinase pathway more efficiently than the common oncogenic p.G12V variant. Thus, also the specific alteration found in this patient could possibly be associated with resistance to anti-EGFR therapy.

Together, these data showed the clinical necessity to investigate the presence of rare or complex *KRAS* variants in mCRC patients before anti-EGFR therapy decision. Moreover, the occurrence of these allelic variants has certainly been underestimated, because many laboratories or commercial *KRAS* detection solutions focus only on the analysis of seven hotspots corresponding to amino-acid changes G12D, G12V, G12C, G12S, G12A, G12R and G13D. Particularly, these additional mutations have an impact on both therapeutic decision-making and molecular testing. We believe that in the absence of (i) *KRAS* standardised testing procedures and (ii) sufficient data on the functional role of rare variants, a comprehensive analysis to identify all *KRAS* mutations in tumour samples is required.

At the present time, no ideal mutation testing method is in use universally for *KRAS* status determination (Jimeno *et al*, 2009). To circumvent *KRAS* mutation targeting, we propose a two-step diagnostic approach. First, an exhaustive analysis through genetic screening of exon 2 and 3 could be carried out by HRM (Wittwer *et al*, 2003). Then, for exons with an altered melting profile, characterisation of the *KRAS* variants by sequencing could be performed. In addition, the use of traditional PCR could be replaced by coamplification at lower denaturation temperature-PCR (Li *et al*, 2008) to reach the detection of low-level somatic mutations that characterise heterogeneous or stromal contaminated samples.

We therefore suggest taking into account the occurrence of rare *KRAS* gene substitutions or more complex alterations for a more accurate patient selection for anti-EGFR therapies. A comprehensive detection of these alterations is not an inaccessible summit. The rapid, reliable, comprehensive and cost-limited two-step approach we propose is relatively easy to implement without any waste of time or money.
